# EphA4‐deleted microenvironment regulates cancer development and leukemoid reaction of the isografted 4T1 murine breast cancer via reduction of an IGF1 signal

**DOI:** 10.1002/cam4.670

**Published:** 2016-02-29

**Authors:** Xuefeng Jing, Takashi Sonoki, Masayasu Miyajima, Takahiro Sawada, Nanako Terada, Shigeki Takemura, Kazushige Sakaguchi

**Affiliations:** ^1^ Departments of Molecular Cell Biology and Molecular MedicineInstitute of Advanced MedicineWakayama Medical UniversitySchool of Medicine811‐1 KimiideraWakayama641‐8509Japan; ^2^ Departments of Hematology/OncologyUniversity HospitalWakayama Medical UniversitySchool of Medicine811‐1 KimiideraWakayama641‐8509Japan; ^3^ Laboratory Animal CenterWakayama Medical UniversitySchool of Medicine811‐1 KimiideraWakayama641‐8509Japan; ^4^ Department of HygieneWakayama Medical UniversitySchool of Medicine811‐1 KimiideraWakayama641‐8509Japan

**Keywords:** 4T1 breast cancer, EphA4, G‐CSF, IGF1, leukemoid reaction

## Abstract

EphA4 belongs to the largest family of receptor tyrosine kinases (RTKs). Although EphA4 is highly expressed in the central nervous system, EphA4 has also been implicated in cancer progression. Most of the studies focus on the expression and function in tumor cells. It is unknown whether EphA4‐deleted microenvironment affects tumor progression. Some of cancers in animals and humans, such as 4T1 cancer cells, are known to produce a large amount of granulocyte colony‐stimulating factors (G‐CSF/Csf3) which can stimulate myeloproliferation, such as myeloid‐derived suppressor cells (MDSCs) leading to a poor recipient prognosis. We isografted 4T1 breast cancer cells into both EphA4‐knockout and control wild‐type female littermate mice. The results showed that the EphA4‐deleted host could inhibit primary tumor growth and tumor metastasis mainly by decreasing the amount of IGF1 synthesis in the circulation and locally tissues. The EphA4‐deleted microenvironment and delayed tumor development reduced the production of G‐CSF resulting in the decrease of splenomegaly and leukemoid reaction including MDSCs, which in turn inhibit the tumor progression. This inhibition can be reversed by supplying the mice with IGF1. However, an excess of IGF1 supply over demand to the control mice could not further accelerate the tumor growth and metastasis. A better understanding and re‐evaluation of the main role of IGF1 in regulating tumor progression could further enhance our cognition of the tumor development niche. Our findings demonstrated that EphA4‐deleted microenvironment impairs tumor‐supporting conditions. Conclusion: Host EphA4 expression regulates cancer development mainly via EphA4‐mediated IGF1 synthesis signal. Thus, targeting this signaling pathway may provide a potential therapeutic option for cancer treatment.

## Introduction

In the past decades, the major focus of cancer research has been on the transformed cells themselves. However, new evidence showed that the interaction between tumor cells and their nontumor milieu plays a critical role during tumorigenesis and tumor progression. Ephrin receptors (Ephs) belong to a superfamily of receptor tyrosine kinases classified into two subclasses, A and B, by their ligand‐binding specificity 1. Among the Ephs, EphA4 binds to both ephrin‐As and ephrin‐Bs 2. Eph/ephrin expression is altered in many cancers leading to changes in cancer cell proliferation, adhesion, cytoskeleton, migration, and survival 3, 4, 5. Ephs and ephrins are expressed in both adult epithelial tissues and stromal cells, including the cells with myeloid phenotypes, where their roles are beginning to be elucidated 5, 6, 7, 8. Evidence further demonstrated that Eph/ephrin signaling plays multifaceted and controversial roles in several kinds of cancer including breast cancer 9, 10. However, there is limited information on the effects of EphA4 on tumor microenvironment, which includes the surrounding support connective tissue, hormones, immunoreaction cells, and other humoral growth factors for tumor progression.

The growth hormone (GH) and insulin‐like growth factor 1 (IGF1) axis plays an important role in normal and pathological development which include cancer 11, 12, 13. Recently, we reported that functions of EphA4 in JAK2‐dependent and ‐independent STAT5B activation leading to enhanced synthesis of IGF1. The deletion of EphA4 expression decreased the amount of IGF1 in the circulation and locally in tissues, which lead to the delayed body growth and development 14. We hypothesized that the EphA4‐deleted host may be able to delay the tumor progression.

Granulocyte colony‐stimulating factor (G‐CSF) is a cytokine produced by macrophages, fibroblasts, and endothelial cells. A cancer‐associated leukemoid reaction with extramedullary hematopoiesis (EMH) and splenomegaly could be induced by G‐CSF which was released by a variety of tumors, including breast cancers in animals and humans 15, 16, 17, 18. G‐CSF appears to promote cancer progression either directly by STAT3 signal 19 or indirectly by stimulating myeloid‐derived suppressor cells (MDSCs) proliferation 20, 21. Therefore, it was considered that cancer‐associated G‐CSF is a cancer stem cell‐specific growth factor 19. G‐CSF or myeloproliferation may represent a novel therapeutic target in cancer 15. A recent study confirmed that poorly immunogenic 4T1 murine metastatic cancer could be eradicated through reducing tumor‐associated circulating MDSCs 22. However, the authors did not mention the impact of tumor‐released G‐CSF which stimulated MDSC proliferation. The causal relationship with G‐CSF remains unclear because eliminating G‐CSF resulted in the removal of tumor‐associated circulating MDSCs. The key point in therapeutic target could be to potentially block the vicious cycle where tumor cells produce large amount of G‐CSF leading to myeloproliferation including MDSCs, which in turn promote tumor growth and progression. Hence, the purpose of this study is also to determine if EphA4‐deleted microenvironment affects circulation level of G‐CSF.

## Materials and Methods

### Cell line, cell culture, and morphology analysis

4T1 breast cancer cell line was made by Fred Miller (Wayne State University School of Medicine, USA) 23, and was kindly provided by Jin Chen (Vanderbilt University School of Medicine, USA) 24. 4T1 cancer cells were cultured and maintained in Dulbecco's modified Eagle's medium (DMEM) supplemented with 10% fetal bovine serum and 5 U/mL penicillin–streptomycin mixture. The morphologic characteristic of the cell line was analyzed and photographed by using Nikon headstand light microscope.

### EGFP in 4T1 cell line

In order to recognize the tumor cells easily, we used a pMXs‐IG vector (obtained from Toshio Kitamura, University of Tokyo, Tokyo, Japan) linked to Expression of enhanced green fluorescent protein (EGFP) through the internal ribosomal entry site (IRES) sequence and incorporated into retrovirus in the experiments as described previously 25. The retrovirus particles were resuspended in the culture medium for 4T1 cells to be transduced. The transduced cells were selected and identified by visualization of EGFP fluorescence.

### Breast cancer models of EphA4‐deleted mice

Generation of EphA4‐deficient mice was described previously 14 The targeted allele was placed on a BALB/c genetic background (Wakayama Medical University Animal Care Center, Wakayama Medical University, Wakayama, Japan) for tumor cell transplantation. The left inguinal mammary fat pad was exposed by dissection. We separately isografted either 10^5^ 4T1 tumor cells or 10^5^ 4T1‐EGFP tumor cells in 30 *μ*L serum‐free DMEM into the exposed left inguinal mammary fat pad to make the murine breast cancer models. The mice used in this study were genotype matched for both EphA4‐KO and control EphA4‐WT female littermate mice at 9–11 weeks of age.

### IGF1 treatment

In order to examine the effect of IGF1 treatment on the tumor development in both EphA4‐KO and control wild‐type (WT) mice, recombinant human IGF1 (5 mg/kg body weight (BW)/day 14 was subcutaneously injected into the EphA4‐KO mice for 9 weeks starting 4 weeks before grafting 4T1 cells. The corresponding WT control mice were treated with either IGF1 or saline for the same period. The dose of IGF1 was decided by series dose (from low to high) experiments as described in our previous study 14. We used the maximum dose of IGF1 for the experiments because the IGF1 supply past this dosage did not work.

### Tumor growth and tumor metastasis analysis

EphA4‐KO and control WT tumor‐bearing littermate mice were killed during the period between fifth and seventh week after transplant and the primary tumors were isolated and weighed. We evaluated the extent of metastasis by counting the number of metastatic foci larger than one millimeter in diameter in the organs of lung, heart, liver, kidney, adrenal glands, peritoneum, pleura, and ovary. All of the organs including tumors were fixed in 10% neutral buffered formalin solution (Sigma‐Aldrich) and metastatic nodules were counted from at least six pairs of littermate mice. The reasons for us to collect the samples at different time points between fifth and seventh week after transplant were as follows: (1) in our preliminary experiments, we found that the isografted primary tumor could be identified in 2 weeks and became apparent 3 weeks after tumor cells were transplanted into the mammary. PBL (peripheral blood leukocyte) increase usually started from the fourth week after transplant; (2) the tumor‐bearing mice developed illness usually after the fifth week of transplant, their condition worsened and they became exhausted after the seventh week of transplant; (3) we were interested in the pathologic changes at different time points during the illness period.

### PBL counts and morphology

Peripheral blood leukocyte counts and smear films were made on a weekly basis from the tail blood obtained by cutting the end of tail and drawn in a heparinized tube. The number of PBL was determined by Particle Counter PCE‐310 (ERMA Inc. Japan Tokyo). The morphological analysis was made on Giemsa‐stained smears.

### Splenomegaly, medullary and EMH analysis

Splenomegaly was analyzed by weighing the spleen weight. Paired EphA4‐KO and control ‐WT tumor‐bearing littermate mice were killed during the period between fifth and seventh week after transplant and spleens were weighed. The same age normal mice without tumor cell transplant (tumor‐free) EphA4‐KO and EphA4‐WT mice were also experimented and spleens were weighed as normal control. Hematopoietic analysis of femur bone marrow (BM), spleen, and liver were performed by paraffin embedded and hematoxylin and eosin (HE) staining sections. Splenic EMH was quantified as the reduction of lymphocyte‐rich white pulp area of spleen on paired EphA4‐KO and ‐WT mice in three low‐power fields per mouse and expressed as percentage of the lymphocyte‐rich white pulp area. Liver EMH was quantified in five high‐power fields per mouse to count the number of myeloid cell precursors and expressed as the average number per field. The histological quantification was performed using the KEYENCE BZ‐X image quantification analyzer.

### ELISA assay

Plasma samples were collected when the two genotypic mice of EphA4‐KO and EphA4‐WT were killed as mentioned above. The plasma samples were stored at −80°C for use. Plasma G‐CSF and IGF1 concentration was examined by ELISA assay (Mouse ELISA Kit ab100684‐G‐CSF abcam and Mouse/Rat IGF‐I Quantikine ELISA Kit R&D Systems).

### Statistical analysis

All statistical analyses were performed with six pairs of each genotype. The paired mice parameters were first analyzed with Student's *t*‐tests and individual treatment groups were analyzed with Tukey's multiple comparison test. *P* value of less than 0.05 was considered significant. All of the above experiments were repeated more than twice.

## Results

### EphA4‐deleted host decreased tumor growth and metastasis

**Figure 1:**
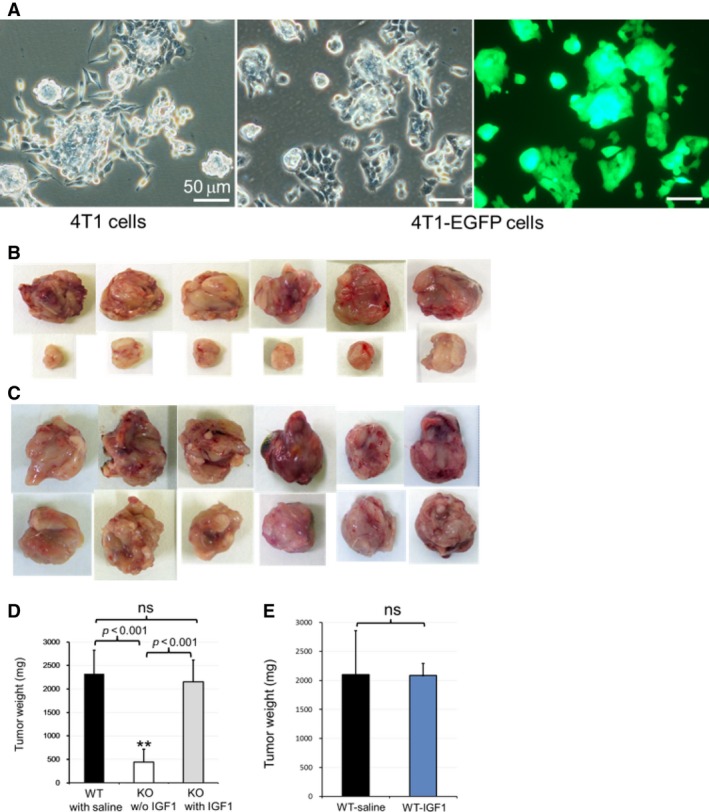
EphA4 deficiency impaired 4T1 stem‐like cancer cell growth niche. (A) cell morphology showed that 4T1 and 4T1‐ Expression of enhanced green fluorescent protein cells were clustered forming cell‐spheres rather than single layer of cells, which was considered stem‐like cells with high malignant potential. (B) the tumors dissected from six paired 5–7 week tumor‐bearing littermate mice (The upper lane: from EphA4‐WT tumor‐bearing control mice; the lower lane: from EphA4‐KO tumor‐bearing mice w/o IGF1 administration). (C) the tumors dissected from six paired 5–7 week tumor‐bearing littermate mice (The upper lane: from EphA4‐WT tumor‐bearing control mice; the lower lane: from EphA4‐KO tumor‐bearing mice with IGF1 administration). (D) statistical analysis showed significant difference of tumor weight between the paired EphA4‐KO w/o IGF1 treatment and WT‐control tumor‐bearing littermate mice. When treated with IGF1, EphA4‐KO mice showed significant weight gain of the primary tumors almost comparable to the WT level (WT:* n *= 12; KO:* n* = 6 of each treatment. Tukey's multiple comparison test). (E) there is no significant difference of tumor weight between control EphA4‐WT mice with saline treatment and that with IGF1 treatment.

It was reported that 4T1 tumor cell line was derived from a spontaneously arising mammary tumor in BALB/c mice and has high malignant potential 26. The cell morphology showed that 4T1 or 4T1‐EGFP tumor cells were clustered forming cell spheres rather than single layer of cells, which were thought to be more malignant potential and considered to be stem‐like cells (Fig.1A). In our preliminary experiments of making metastatic murine breast cancer models by using either 4T1 or 4T1‐EGFP tumor cells separately (more than ten pairs of EphA4‐KO and control WT mice for each cell line), both 4T1 and 4T1‐EGFP tumor cells can be used to make metastatic murine breast cancer models successfully. The 4T1‐EGFP cell line displayed tumor growth and metastatic properties similar to and more stable than that of the parental cell line. The survival time of the models was stabilized by using 4T1‐EGFP cell line transplantation compared to the 4T1 parental cell line. About a quarter of the mice with 4T1 cell transplant died early of tumor‐associated blood ascites in the preliminary experiments. Therefore, we used 4T1‐EGFP cell line for this study.

The isografted primary tumor could be identified 2 weeks and became apparent 3 weeks after tumor cell transplantation. PBL increase usually started from the fourth week after transplant. Thus, we made the dissection for primary tumors isolation, tumor weight measurement, and counting the metastatic tumor foci between the fifth and seventh week of tumor‐bearing EphA4‐KO and ‐WT paired littermate mice. We decided to check the tumor growth by weighing the tumor weight rather than measuring the volume as the tumor shape was irregular and a small difference of the measured width may lead to a big effect of the volume. In the absence of IGF1 administration, the growth retardation of the primary tumor was observed in EphA4‐KO tumor‐bearing mice compared to that of control ‐WT tumor‐bearing littermate mice (Fig. 1B). Significant tumor weight gain was identified in the IGF1 injected EphA4‐KO tumor‐bearing mice. EphA4‐KO mice with IGF1 administration showed significant tumor weight gain to almost the WT level (Fig. 1C and D). However, IGF1 administration did not significantly further increase the tumor weight of the control EphA4‐WT mice (Fig. 1E).

**Figure 2:**
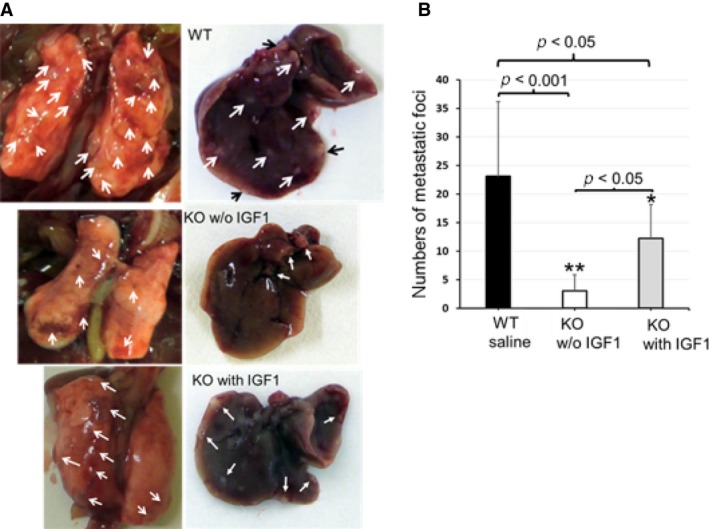
Host EphA4 deficiency reduced metastasis. (A) tumor metastatic foci in the lung (left) and liver (right) in 46‐day control EphA4‐WT tumor‐bearing mouse in the upper lane, paired EphA4‐KO tumor‐bearing littermate mouse without IGF1 treatment in the middle lane and 46‐day EphA4‐KO tumor‐bearing mouse with IGF1 treatment in the lower lane. (B) statistical analysis showed in the absence of IGF1 treatment, number of metastatic tumor foci were significantly reduced in EphA4‐KO mice compared with control WT tumor‐bearing littermate mice (*P < 0.05; **P < 0.001). When treated with IGF1, EphA4‐KO mice experienced a large increase in the number of metastatic tumors (WT:* n* = 12; KO:* n* = 6 of each treatment. Tukey's multiple comparison test).

Metastatic tumors were found in almost all of the organs including lung, liver, heart, spleen, pancreas, kidney, adrenal gland, pleura, peritoneum, and ovary, which were naturally in the end stage (verge of death) of the tumor‐bearing mice. In this study, the tumor metastatic foci were examined on all of the mentioned organs and the foci larger than one millimeter in diameter were counted. In the absence of IGF1 administration, EphA4‐KO 4T1 tumor‐bearing mice showed significantly reduced tumor metastatic foci compared with that in control WT littermate mice (Fig. 2A and B). The number of metastatic tumors was significantly increased in IGF1 administrated KO tumor‐bearing mice, but the IGF1 treatment was unable to enhance the number of metastatic foci to the WT level (Fig. 2B). We also tested the effect of IGF1 treatment for the tumor metastasis of control WT tumor‐bearing mice, but the IGF1 treatment did not significantly further increase the metastatic foci (data not shown).

### EphA4‐deleted host reduced cancer‐related splenomegaly and leukemoid reaction

**Figure 3:**
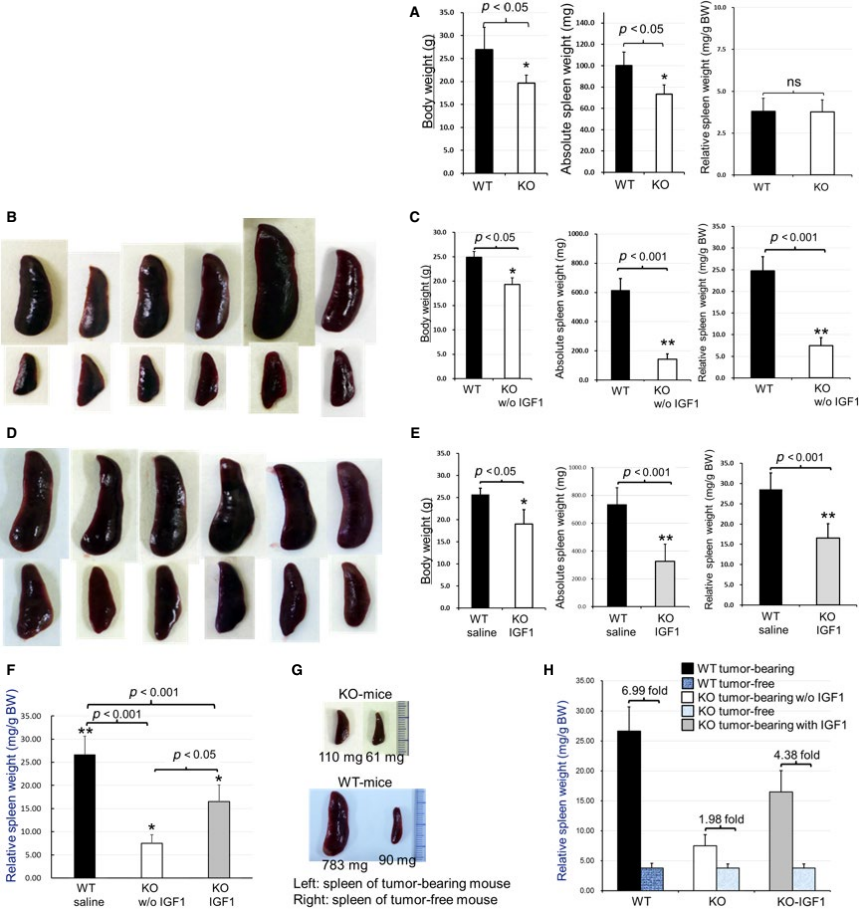
Host EphA4 deficiency reduced splenomegaly. (A) spleen weight was adjusted in relative to body weight of six paired tumor‐free EphA4‐KO and ‐WT mice (the same age as that of tumor‐bearing mice) and showed no significant difference after the adjustment between the two genotypes. (B,C) in the absence of IGF1 treatment, reduced splenic enlargement was found in EphA4‐KO tumor‐bearing mice compared with control ‐WT tumor‐bearing littermate mice (*P < 0.05; **P < 0.001). (B) upper lane showed dissected spleens from 5–7 week tumor‐bearing ‐WT mice; lower lane showed the spleens from 5–7 week tumor‐bearing littermate ‐KO mice without IGF1 treatment. (C) statistical analysis of B (*P < 0.05; **P < 0.001). (D) IGF1 treatment increased the spleen enlargement of EphA4‐KO tumor‐bearing mice (upper lane: from WT tumor‐bearing mice; lower lane: from littermate ‐KO tumor‐bearing mice with IGF1 treatment). (E) statistical analysis of D (*P < 0.05; **P < 0.001). (F) statistical analysis showed in the absence of IGF1 treatment, EphA4‐deleted tumor‐bearing hosts significantly reduced the splenomegaly (*P < 0.05; **P < 0.001), which was markedly enhanced by IGF1 treatment in EphA4‐KO tumor‐bearing mice; however, it could not reach the level of WT (*n* = 12 of ‐WT tumor‐bearing mice and *n* = 6 of ‐KO tumor‐bearing mice each treatment). (G) showed the comparison of spleens between tumor‐bearing and tumor‐free mice of paired EphA4‐KO and ‐WT genotype. (H) statistical analysis showed the degree of increase in spleen weight between tumor‐bearing and tumor‐free mice of paired EphA4‐KO and ‐WT genotype. The EphA4‐WT of tumor‐bearing mice showed increased splenic enlargement close to sevenfold while the ‐KO less than twofold, and IGF1 treatment enhanced multiple times.

Since the EphA4‐KO mice are smaller than the control WT mice 14, we adjusted the spleen weight by body weight. The relative spleen weight showed no significant difference between EphA4‐KO and ‐WT tumor‐free mice (Fig. 3A). In the absence of IGF1 administration, spleen enlargement was significantly decreased in EphA4‐KO tumor‐bearing mice (Fig. 3B and C), and IGF1 treatment was able to markedly increase splenic enlargement of EphA4‐KO tumor‐bearing mice. However, the IGF1 treatment could not enhance splenomegaly to the WT level (Fig. 3D–F). Compared with either the spleen of tumor‐free mice or the spleen of EphA4‐KO tumor‐bearing mouse without IGF1 treatment, the splenic enlargement was conspicuous in the control EphA4‐WT tumor‐bearing mice (Fig. 3G). Although splenic enlargement can be found in both WT and KO tumor‐bearing mice compared with tumor‐free mice, respectively, the splenic enlarged degree was different between the two genotype mice. The degree was close to sevenfold in EphA4‐WT mice, but less than twofold in EphA4‐KO mice and IGF1 treatment enhanced multiple times (Fig. 3H).

**Figure 4:**
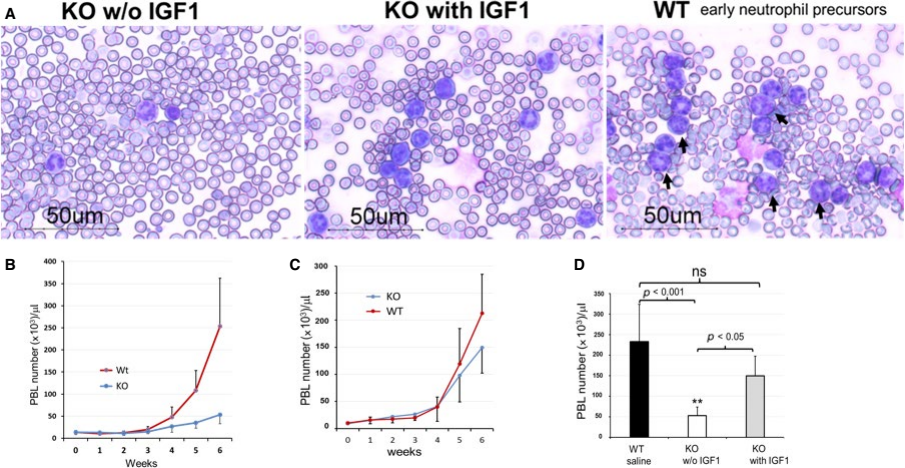
Host EphA4 deficiency reduced leukemoid reaction. (A) EphA4‐KO tumor‐bearing mice without IGF1 treatment (left lane) showed decreased peripheral blood leukocyte (PBL) and neutrophil precursors in the blood smear, which were enhanced by IGF1 treatment (middle lane). EphA4‐WT tumor‐bearing mice showed markedly increased granulocytes and neutrophil precursors (right lane: black arrows showed neutrophil precursors in different stages). (B) in the absence of IGF1 treatment, PBL was markedly reduced in the EphA4‐KO tumor‐bearing mice. (C) EphA4‐KO tumor‐bearing mice with IGF1 treatment showed an enhanced PBL almost to the level of WT mice. (D) multiple comparison test showed the significant increase to the WT level (*n* = 12 of WT tumor‐bearing mice and *n* = 6 of KO tumor‐bearing mice each treatment. **P < 0.001 or *P* < 0.005). Blood samples were collected from six‐week tumor‐bearing mice.

Cancer‐associated leukemoid reaction has been defined as PBL >50,000/L. In the control EphA4‐WT tumor‐bearing mice, the markedly PBL increase usually started from the fourth week after transplant. Severe cancer‐associated myeloproliferation has been found in control WT tumor‐bearing mice when tumor locally advanced or metastatic, which could be reflexed in a quantitative change of increased PBL number and qualitative change of early neutrophil precursors in peripheral blood smears (Fig. 4A). The number of PBL significantly reduced in EphA4‐KO tumor‐bearing mice without IGF1 administration (Fig. 4B), but EphA4‐KO mice with IGF1 treatment showed an enhanced PBL number almost to the level of WT mice (Fig. 4C and D).

### EphA4‐deleted host reduced cancer‐related EMH

**Figure 5:**
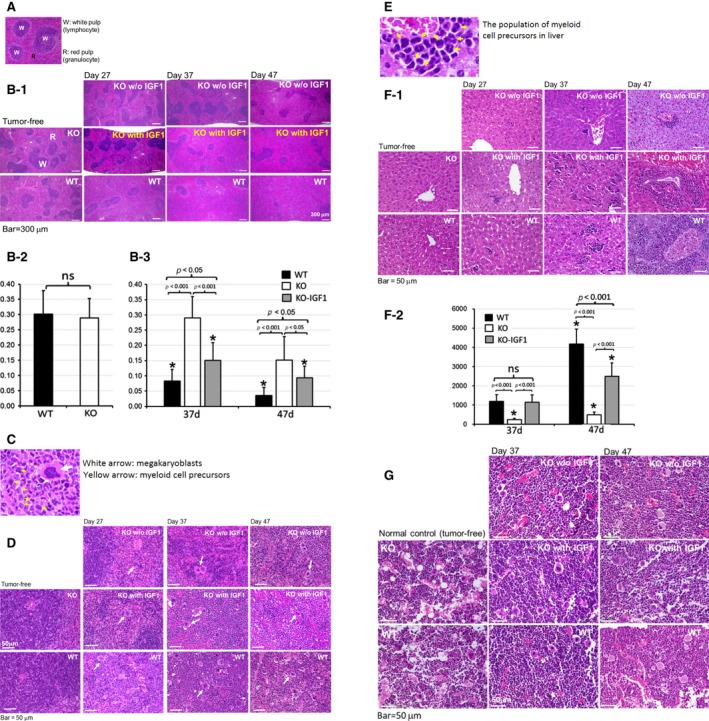
Microscopic images of spleen, liver, and bone marrow (BM) showed EphA4 deficiency reduced cancer‐related myeloproliferation. (A) microscopic image of white and red pulp of normal spleen. (B‐1) low magnification of the spleen with the expanded granulocyte‐rich red pulp and reduction of lymphocyte‐rich white pulp area on day 27, 37, and 47 of EphA4‐KO with or without IGF1 administration and control ‐WT tumor‐bearing mice. EphA4 deficiency reduced the splenic extramedullary hematopoiesis (EMH), which was enhanced by IGF1 treatment. (B‐2) histological quantification of the lymphocyte‐rich white pulp area percentage on tumor‐free mice (*n* = 4 each genotype). (B‐3) histological quantification of the lymphocyte‐rich white pulp area percentage on paired EphA4‐KO and ‐WT tumor‐bearing mice (*n* = 4* for each genotype and treatment, *the samples were collected on the indicated day or closely before or after the indicated day of Figure 5B‐3). (C) higher magnification showed the megakaryoblasts (white arrow) and myeloid cell precursors (yellow arrow). (D) EMH of megakaryoblasts and myeloid cell precursors of Figure 5B‐1 in higher magnification. (E) higher magnification showed myeloid cell precursors of liver. (F‐1) extramedullary hematopoiesis (EMH) was very rare in the liver of EphA4‐KO without IGF1 treatment (myeloid cell precursors were scarce in the liver of EphA4‐KO tumor‐bearing mice on day 27), but markedly enhanced when given IGF1 treatment on day 37 and 47 of tumor‐bearing mice. (F‐2) histological quantification of EMH cells, average number per high‐power field in the liver of paired EphA4‐KO and ‐WT tumor‐bearing mice. (*n* = 4* for each genotype and treatment, *the samples were collected on the indicated day or closely before or after the indicated day of Figure 5F‐2). (G) higher magnification of hematoxylin and eosin (HE)‐stained femur bone marrow (BM) section showed accumulation of myeloid cells at various stages of differentiation. Although cancer‐associated myeloproliferation of BM was identified on day 37 and 47 after tumor cell transplant, we could not be differentiated from EphA4‐KO tumor‐bearing mice with or without IGF1 treatment and control ‐WT tumor‐bearing mice.

It is considered that severe leukemoid reaction was caused by both intra‐ and extramedullary hematopoiesis. Besides the examination of the PBL number and blood smears, we also examined the hematopoietic condition of BM, spleen, and liver. The PBL number began to increase from the fourth week of tumor cell transplantation, which reflected myelopoiesis and EMH. The femur bone, spleen, and liver were isolated and fixed at different day (day 27, day 37, and day 47) (Fig. 5) after tumor cells transplant. The hematoxylin and eosin (HE) stained spleen sections showed the severe EMH of expanded granulocyte‐rich red pulp with reduction in white pulp area (lymphocytes) in the control EphA4‐WT tumor‐bearing mice (Fig. 5A–D). These changes were reduced in EphA4‐KO tumor‐bearing mice without IGF1 administration and enhanced by IGF1 treatment (Fig. 5B‐1–3), although the IGF1 treatment was unable to enhance the change to the WT level (Fig. 5B‐3). Higher magnification of the HE‐stained sections showed prominent megakaryoblasts and myeloid cell precursors in the splenic red pulp of tumor‐bearing mice (Fig. 5C, D), which reflected EMH. These changes were reduced in EphA4‐KO tumor‐bearing mice without IGF1 treatment compared to that of control WT tumor‐bearing mice and could be enhanced in EphA4‐KO tumor‐bearing mice with IGF1 treatment. Myeloid cell precursors of EMH were very rare or markedly reduced in the liver of EphA4‐KO tumor‐bearing mice without IGF1 treatment, but markedly increased with IGF1 administration (Fig. 5E, F‐1, F‐2). The sections of BM showed cancer‐associated myelopoiesis in both EphA4‐KO tumor‐bearing mice without or with IGF1 treatment and control WT tumor‐bearing mice (Fig. 5G). It was hard to differentiate the BM image between the two genotypes of tumor‐bearing mice.

### Plasma levels of cancer‐related G‐CSF and IGF1 were reduced in EphA4‐deleted mice

**Figure 6:**
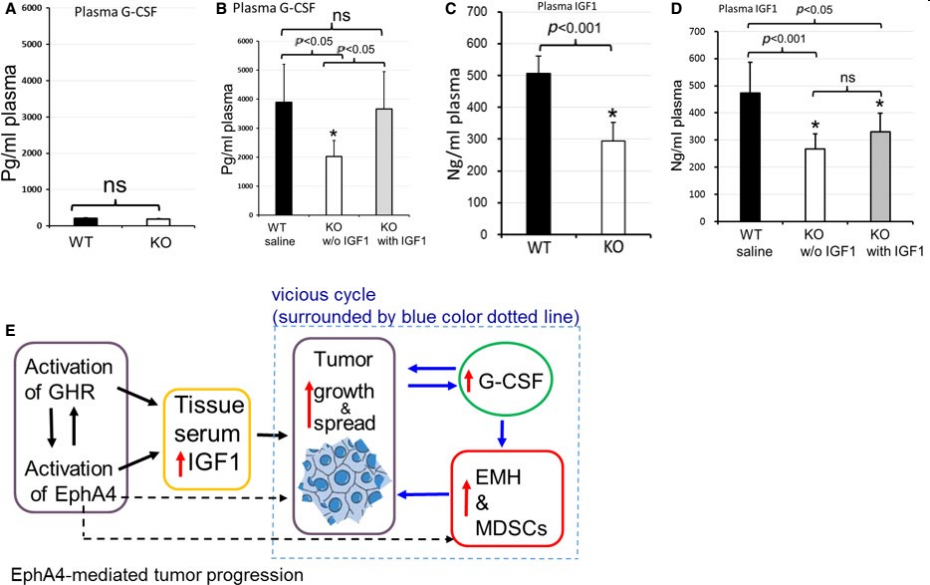
Cancer‐related circulation G‐CSF was reduced by EphA4 deficiency. (A) G‐CSF ELISA assay showed that plasma G‐CSF level was very low and there was no significant difference between the two genotypes of tumor‐free mice. (B) EphA4‐KO tumor‐bearing mice without IGF1 administration showed a significant reduction in G‐CSF circulation level, which was enhanced by IGF1 treatment up to a level similar to that observed in WT tumor‐bearing mice. (WT:* n* = 12; KO:* n* = 6 of each treatment of 5–7 week paired tumor‐bearing mice). (C) The level of IGF1 was significantly less in EphA4‐KO tumor‐free mice compared with ‐WT tumor‐free mice (*n* = 6 of each genotype of tumor‐free mice). (D) IGF1 treatment in EphA4‐KO mice was unable to maintain IGF1 concentration in the blood to the WT level of an endogenous IGF1, although the treatment could enhance the tumor growth. (WT:* n* = 12; KO:* n* = 6 of each treatment of 5–7 week paired tumor‐bearing mice). (E) The vicious cycle (surrounded by blue dotted line) was regulated by EphA4‐mediated IGF1 synthesis pathway. The vicious cycle means that IGF1 increases tumor growth which enhanced plasma G‐CSF level. The increased G‐CSF level stimulates the myeloproliferation and extramedullary hematopoiesis (EMH) leading to a production of myeloid‐derived suppressor cells (MDSCs), which in turn promotes tumor growth and metastasis. Host EphA4 deficiency impaired the tumor progression vicious cycle mainly via decreasing IGF1 synthesis, and others (black dotted lines) such as FGFR pathway (mentioned in context) and unknown molecules.

We next analyzed plasma G‐CSF level by ELISA because G‐CSF was thought to be released by 4T1 tumor cells and often elevated in tumor‐bearing host with potent activities to stimulate EMH and MDSC proliferation. We found the circulation G‐CSF level was very low in tumor‐free mice and no significant difference between EphA4‐KO and WT mice at the same age as that of tumor‐bearing mice (Fig. 6A). A significant rise of circulation G‐CSF level more than 18‐fold of WT genotype and 10‐fold of KO genotype was observed in the tumor‐bearing mice compared with that of tumor‐free mice. EphA4‐KO tumor‐bearing mice without IGF1 administration showed a significant reduction in G‐CSF circulation level, which was enhanced by IGF1 treatment up to a level similar to that observed in WT tumor‐bearing mice (Fig. 6B). To investigate the effect of IGF1 treatment and tumor growth on plasma level of IGF1, we analyzed plasma IGF1 level of tumor‐bearing mice compared with tumor‐free mice by ELISA assay. The plasma level of IGF1 was significantly lower in Epha4‐KO tumor‐free mice compared with ‐WT tumor‐free mice (Fig. 6C).Similar results were found in tumor‐bearing mice (Fig. 6D). IGF1 treatment showed slightly increased IGF1 level in EphA4‐KO tumor‐bearing mice but the increase was not statistically significant. While IGF1 treatment could enhance tumor growth, it was unable to maintain IGF1 concentration in the blood at the WT level of an endogenous IGF1. The blood samples were collected at the end of IGF1 treatment which may be another reason for the relatively low IGF1 level.

## Discussion

The interactions between tumor malignant potential and host individual differences are crucial for tumor growth and progression 27, 28. In this study, we provided the novel evidence that host EphA4 deficiency inhibited murine breast cancer growth and metastasis by reducing the EphA4‐GH receptor (GHR)‐IGF1 and/or EphA4‐IGF1 activities, which lead to decreasing IGF1 production. The inhibition caused by host EphA4 deficiency could be retrieved by IGF1 supplement. The mechanism of EphA4 regulating IGF1 synthesis was reported in our previous study 14, in which we demonstrated the interaction of EphA4 with GHR, Janus kinase 2 (JAK2), and signal transducers and activators of the transcription 5B (STAT5B). The interaction resulted in enhanced JAK2/STAT5B activation in response to the stimulation by ephrinA1 and GH, leading to the augment of JAK2/STAT5B‐dependent Igf1 mRNA expression (GHR/EphA4/JAK2/STAT5B). EphA4 is also an important component of the GHR signaling pathway which leads to IGF1 synthesis without affecting the expression of GHR (EphA4/STAT5B) 14. These interactions of tumor cell development niche and tumor cell malignant potential steered the progression of the cancer.

### EphA4‐deleted host impaired the cancer stem cell growth niche

Malignant tumor is heterogeneous with substantial genotypic and phenotypic diversity. It was reported that 67NR, 4T07, and 4T1 tumor cell lines were derived from one spontaneously arising mammary tumor in BALB/c mouse, but have different malignant potential 26. Compared with 67NR and 4T07 cell lines, 4T1 tumor cells represent a subset of tumor malignancy with stem‐like properties and phenotype, which can release tumor growth stimulators and generate a suitable tumor growth niche for tumor cells to evade from host immunosurveillance. Hence, the emerging topic is the importance of a stem‐like niche in regulating the biological properties of stem‐like cancer cells. The interactions between cancer cells and individual different niche composition is crucial for tumor growth and metastasis. Our study focused on the tumor growth niche by using a different genetic background of the host such as EphA4‐KO and WT to analyze the regulation mechanism of stem‐like niche. Many of the elements in the niche, including the innate and adaptive cells and factors, play multifaceted roles during cancer development and can promote or inhibit tumor progression, depending on local and systemic conditions. Our results indicate that the EphA4‐deleted host can potentially change the crosstalk of humoral and cell–cell contact‐mediated signals in cancer development and play a critical role to inhibit the growth of even more malignant stem‐like cancer cells.

### EphA4‐deleted host impairs tumor‐supporting condition mainly by inhibiting IGF1 production

We have previously demonstrated that EphA4 can enhance cell proliferation, differentiation, and migration either by cross‐talking with fibroblast growth factor receptor (FGFR) 25 or via GHR/EphA4/JAK2/STAT5B or/and/EphA4/STAT5B signal pathway IGF1 synthesis 14. Despite the accumulating evidence that indicates Eph family plays an important role in every step of tumor progression, including tumor growth, angiogenesis, invasion, and metastasis in humans and mice 4, 9, most of the studies focused on the expression and function in tumor cells. The mechanism on how EphA4 affected host environment to regulate tumor growth and progression is still unknown. The results we obtained may provide novel information on the mechanisms of host EphA4‐deficiency inhibiting tumor progression mainly via EphA4‐mediated IGF1 production. Our data are consistent with other studies that showed reduced circulating IGF1 levels can delay breast tumor onset and growth in IGF1‐deficient mice compared to WT mice 29. In humans, Laron‐type dwarfism with low IGF1 levels is associated with low cancer risk 30.

Our findings confirmed that IGF1 administration could enhance the tumor growth of EphA4‐deleted mice up to a level similar to that observed in control WT mice, but could not enhance the metastatic tumor foci of EphA4‐deleted mice up to the WT level. Against our expectation, administration of IGF1 was unable to further increase the tumor growth and metastasis in the control WT mice, suggesting that an excess of IGF1 supply over demand to the control mice could not accelerate the tumor progression. In fact, IGF1 was unable to induce oncogenic transformation, but can help sustain the survival of transformed cells mainly by overriding the signals of apoptosis 31. IGF1/IGF1R signaling was not able to enhance metastasis directly but via inducing tumor‐associated lymphangiogenesis contributing to cancer lymphatic metastasis 32 or via tumor‐associated leukemoid reaction resulting in immunosuppression 33. Although increased IGF1 plasma levels were positively associated with poor prognosis of different kinds of cancer including breast cancer 34, 35, the mechanism is still not clear. The possible mechanism may be due to its enhancement of the tumor growth which is essential for tumor progression or/and the tumor‐released regulation molecules. For over a century, a lot of efforts were invested in developing strategies to target IGF1 receptor (IGF1R) in cancer therapy, but accumulating data from clinical study indicated that specific targeting of the IGF1R was not efficient as an anti‐breast cancer therapy 36. Besides IGF1R, targeted deletion of hepatic igf1 in the transgenic adenocarcinoma of the mouse prostate (TRAMP) model could lead to dramatic alterations in the circulating IGF axis, but does not reduce tumor progression, which may require a reduction in GH levels as well 37, 38. In view of this, a better understanding of the limited role of IGF1 or IGF1R in regulating tumor progression will potentially re‐evaluate their functions. Our results demonstrated that the regulation of tumor development by IGF1 was mainly for the enhancement of tumor growth. The murine breast tumor metastasis, splenomegaly, EMH, and leukemoid reaction were not only impacted by tumor growth but also other regulators and signal pathways. EphA4 appears to have multiple pathways in regulating tumor development beyond IGF1 synthesis, such as the EphA4–FGFR pathway 25. Activation of the FGF/FGFR system may lead to neovascularization, metastatic dissemination, and tumor progression 39, 40. Our findings suggested that targeting EphA4 may lead to a novel inhibiting effect combined with other regulators in breast cancer therapy, especially where the tumor cell proliferation and survival are dependent on local tissue IGF1 production.

### EphA4‐deleted host inhibits cancer‐associated myeloproliferation

In recent years, an increasing number of studies has been focusing on the role of cancer‐associated myeloproliferation 15, especially in mammary cancer researches 41. Although it was a paraneoplastic epiphenomenon, the presence of G‐CSF produced by tumor cells is far from incidental on a tumor development. The cancer‐related G‐CSF could stimulate the BM and EMH to produce super large number of MDSCs 22, 42 resulting in severe leukemoid reaction leading to a very poor prognosis 16, 17, 18, 43. A study reported that invasive breast cancer reprograms early myeloid differentiation to generate immunosuppressive neutrophils 44. It demonstrated that tumor‐released prolonged G‐CSF stimulation induces activation of a myeloid differentiation program in bone marrow. However, it is ultimately inefficient to overcome the enlarged spleens to meet the demands of hematopoiesis during tumor progression for both the development and activity of immunosuppressive neutrophils in cancer 44.

Our study showed similar results that splenomegaly is remarkably associated with the high number of PBL (leukemoid reaction) and the large amount of myeloid progenitor cells in the peripheral blood, which was decreased by EphA4 deleted host. Therefore, the significantly enlarged spleen and splenic EMH might be the primary sites of MDSC proliferation in the murine breast cancer. EphA4‐deleted host significantly inhibited G‐CSF‐related splenic EMH, but could not be enhanced to the WT level by IGF1 treatment. There should be some other pathway combined with IGF1 regulating the EMH (myeloproliferation) in EphA4‐deleted microenvironment, such as the EphA4‐FGFR pathway 25, 40.

Evidence has identified that cancer‐associated G‐CSF is responsible for the recruitment of MDSCs and dysregulates hematopoiesis, which promote tumor growth and metastasis via inhibition of antitumor immune responses 42, 45. Increased clinical data also revealed that G‐CSF and SDMCs aggravated cancer deaths 15, 46. Understanding the concepts of regulators and underlying causes involved in cancer‐associated myeloproliferation could enhance our cognition of the importance of its molecular basis. Thus, the study of cancer‐associated G‐CSF is crucial in both human and murine cancers. It has been demonstrated that 4T1 tumor‐associated myeloproliferation increased the number of MDSCs mainly from splenic and hepatic EMH 47. EphA4‐deleted host significantly reduced the splenomegaly resulting in a marked reduction in circulating MDSCs, which may be mainly via reduced tumor growth and decreased G‐CSF releasing. This in turn reduced the number of MDSCs, which could effectively inhibit the tumor development 22. IGF1 administration could enhance the tumor growth almost to the WT level, but could not enhance the splenomegaly to the WT level.

The reason behind why IGF1‐stimulated tumor growth could not produce sufficient G‐CSF for splenic EMH to reach WT level is due to EphA4‐FGFR signaling, which regulates the MDSC proliferation 40. In 4T1 tumor‐bearing mice, the FGFR inhibition by FGFR inhibitor PD173074 reduced MDSCs in the circulation, spleen, and tumor, which significantly inhibited tumor metastasis 40. The decreased metastasis might reduce the G‐CSF releasing. Another reason may be the increased liver EMH in Eph4‐KO tumor‐bearing mice, which can partially meet the demands of hematopoiesis during tumor progression for both the development and activity of MDSCs in cancer. The positive correlation between proliferated leukocytes or MDSCs and tumor volume or G‐CSF/GM‐CSF transcript level was confirmed by other studies 16, 48 which was also consistent with our results that the severe leukemoid reactions were related to the marked increased G‐CSF level induced by the advanced large tumors. A G‐CSF loss and gain‐of‐function study showed tumor‐derived G‐CSF facilitates tumor progression via granulocytic MDSC‐dependent mechanism and reduction of circulating MDSCs eradicated 4T1 tumors 22, 49. In summary, the above data may lead to better understanding of the molecular basis and regulation of tumor‐associated EMH. Thus, the crucial point is to understand how to intervene the vicious cycle of tumor growth releasing G‐CSF which further stimulates MDSCs proliferation that lead to tumor progression. Our findings may provide further information to understand the complexity of the interactions between tumors and their microenvironment (the stem cell niche). As we have already mentioned that host EphA4 regulates tumor progression via multiple signal pathways. The IGF1 synthesis signal is an important one of them. EphA4‐deficient microenvironment could play an important role in impairing the tumor development niche and blocking the vicious cycle (Fig. 6E) to inhibit the tumor progression.

## Conclusions

(1) EphA4‐deleted microenvironment reduced tumor growth and metastasis mainly via reduction in an IGF1 synthesis signal; (2) An excess of IGF1 supply over demand to the control mice could not further accelerate the tumor development; (3) The cancer‐associated leukemoid reaction is far from incidental epiphenomenon, secondary to underlying primary disease and the severe leukemoid reaction is lethal; (4) Targeting host EphA4 expression may serve as a novel candidate in blocking the tumor progression vicious circle (Fig. 6E) for G‐CSF‐releasing or IGF1‐dependent cancer.

## Conflict of Interest

The authors disclose no potential conflicts of interest.
